# Population-based surveillance for congenital zika virus syndrome: a latent class analysis of recorded cases from 2015–2018

**DOI:** 10.1186/s12884-022-04860-3

**Published:** 2022-06-29

**Authors:** Enny S. Paixao, Laura C. Rodrigues, Maria da Conceição N. Costa, Rita de Cassia Oliveira de Carvalho-Sauer, Wanderson K. Oliveira, Luciana L. Cardim, Lavinia Schuler-Faccini, Roberto F. S. Andrade, Moreno S. Rodrigues, Elizabeth B. Brickley, Rafael V. Veiga, Larissa C. Costa, Eduardo H. Carmo, Liam Smeeth, Mauricio L. Barreto, Maria Gloria Teixeira

**Affiliations:** 1 London School of Hygiene and Tropical Medicine, Keppel St, London, WC1E 7HT UK; 2 Center of Data and Knowledge Integration for Health (CIDACS), Gonçalo Moniz Institute, Oswaldo Cruz Foundation, Salvador, Bahia Brazil; 3 Instituto de Saude Coletiva, Federal University of Bahia, Salvador, Bahia Brazil; 4 East Regional Health Center of the State Health Secretariat of Bahia, Santo Antonio de Jesus, Bahia Brazil; 5 Ministry of Defense Hospital das Armed Forces, Technical Directorate of Education and Research, Brasília, Brazil; 6 Genetics Department, Universidade Federal Do Rio Grande Do Sul, Porto Alegre, Rio Grande do Sul Brazil; 7 Medical Genetics Service, Hospital de Clinicas de Porto Alegre, Porto Alegre, Brazil

**Keywords:** Congenital zika syndrome, Mortality, Microcephaly, Imaging findings

## Abstract

**Objective:**

This study aims to describe clinical findings and determine the medium-term survival of congenital zika syndrome (CZS) suspected cases.

**Methods:**

A retrospective cohort study using routine register-based linked data. It included all suspected cases of CZS born in Brazil from January 1, 2015, to December 31, 2018, and followed up from birth until death, 36 months, or December 31, 2018, whichever came first. Latent class analysis was used to cluster unconfirmed cases into classes with similar combinations of anthropometry at birth, imaging findings, maternally reported rash, region, and year of birth. Kaplan–Meier curves were plotted, and Cox proportional hazards models were fitted to determine mortality up to 36 months.

**Results:**

We followed 11,850 suspected cases of CZS, of which 28.3% were confirmed, 9.3% inconclusive and 62.4% unconfirmed. Confirmed cases had almost two times higher mortality when compared with unconfirmed cases. Among unconfirmed cases, we identified three distinct clusters with different mortality trajectories. The highest mortality risk was observed in those with abnormal imaging findings compatible with congenital infections (HR = 12.6; IC95%8.8–18.0) and other abnormalities (HR = 11.6; IC95%8.6–15.6) compared with those with normal imaging findings. The risk was high in those with severe microcephaly (HR = 8.2; IC95%6.4–10.6) and macrocephaly (HR = 6.6; IC95%4.5–9.7) compared with normal head size.

**Conclusion:**

Abnormal imaging and head circumference appear to be the main drivers of the increased mortality among suspected cases of CZS. We suggest identifying children who are more likely to die and have a greater need to optimise interventions and resource allocation regardless of the final diagnoses.

**Supplementary Information:**

The online version contains supplementary material available at 10.1186/s12884-022-04860-3.

## Introduction

After a cluster of microcephaly cases was first reported in Northeast Brazil in 2015 [1, 2], a causal relationship with Zika virus (ZIKV) was suspected [3], and the Brazilian Ministry of Health used the Public Health Events Register (RESP) [4] to record data on all suspected cases of this new condition [5], later called Congenital Zika Syndrome (CZS) [6, 7]. One of the first objectives of RESP was to provide a rapid assessment of the magnitude and geographical distribution of the epidemic and enable an early understanding of the clinical spectrum of the syndrome. For these reasons, the early notification criteria were broad to capture as many cases as possible. As with any new condition, establishing a classification based on clinical-epidemiological criteria is challenging before acquiring knowledge on the natural history of the disease and given the early limitations of diagnostic/testing capacity. The classification of suspected cases relied initially on clinical observation and imaging. The criteria were refined several times during the epidemic as knowledge gaps were filled [5], and many cases notified as suspected were subsequently reviewed.

Recorded suspected cases were thus later classified as (a) Confirmed cases due to aetiological identification (laboratory confirmation for ZIKV in a molecular or serological test of a sample from the mother, fetus, or newborn) or imaging and clinical examination. (b) Probable cases were those with signs or symptoms compatible with the condition but whose mothers did not report symptoms during pregnancy and had a negative or inconclusive result for a specific laboratory test for ZIKV. (c) Unconfirmed cases. Suspected cases were unconfirmed for a variety of reasons: they could have received a different diagnosis (other congenital infections, e.g., syphilis, toxoplasmosis), congenital genetic malformations, foetal alcoholic syndrome, etc. They could also have been unconfirmed because the criteria used to define microcephaly/CZS became stricter and they no longer met the criteria, or because further investigation did not find imaging results consistent with what was then perceived to be characteristic of CZS [8].

Prior studies have described the characteristics of suspected cases recorded in RESP, in 2015 and 2016 [9, 10]. However, these studies did not explore the potential heterogeneity among those unconfirmed cases that may exist, since they could have been notified to the system for different reasons that vary from slightly reduced head circumference (HC), due to the wider criteria used at the beginning of the epidemic, to severe abnormalities described in imaging findings. As it is possible that some of these conditions are life-threatening or result in lifelong impairment, we investigate the medium-term outcomes of suspected cases.

This study is based on national data on all suspected Congenital Zika Syndrome cases in Brazil from 2015–2018. The aims of this study were (i) to describe anthropometry at birth, imaging findings and, mortality up to 36 months of life according to a final epidemiological classification (confirmed/probable, inconclusive or unconfirmed) and (ii) to investigate patterns across a range of characteristics (head circumference, birth weight, imaging tests, place and year of birth) among unconfirmed cases, to identify similar clusters and explore mortality by cluster characteristics up to the age of 36 months. We hypothesise that unconfirmed cases with abnormal anthropometric measures at birth and abnormal imaging findings have an increased risk of death, either because they are cases of atypical CZS or because they have other severe pathologies. In addressing this hypothesis, we aim to identify patterns of clinical features to better understand possible causal pathways towards lethal outcomes, for example, by recognising characteristics that are strongly associated with mortality.

## Methods

### Study design

We conducted a retrospective population-based study, including all suspected cases of CZS registered in RESP and as a comparison group the population of live births without a record of CZS, born in Brazil from January 1, 2015, to December 31, 2018. Both groups were followed up from birth until death, 36 months, or December 31, 2018, whichever came first.

### Data sources

Cases were notified to RESP when considered to have microcephaly and/or central nervous system (CNS) alterations confirmed or suspected to be associated with congenital ZIKV infection since 2015. We obtained data on all notifications to RESP and extracted data on final CZS classification (confirmed/probable, inconclusive or unconfirmed), head circumference, imaging findings, and maternal reporting of a rash during pregnancy [4]. Suspected cases are those notified to RESP. They were notified because they meet one or more of the criteria defined for notification at one point during the period [8]:

1. microcephaly was defined as HC of 33 cm or less for term boys and girls, which was reduced to 32 cm on December 12, 2015, and reduced again (March 2016 following the World Health Organization (WHO) recommendation) to 31.9 for term boys and 31.5 for girls or more than two standard deviations below the mean for age and sex (according to INTERGROWTH 21st standards for those born at fewer than 37 gestation weeks, or WHO standards for those born with 37 gestation weeks or more);
  or
2. central nervous system changes suggestive of congenital infection detected from neuroimaging tests (accepted imaging were cranial computed tomography, brain magnetic resonance, or transfontanellar ultrasound); two or more neurological, visual or auditory manifestations;
  or
3. newborns or fetuses from mothers who reported a fever and/or skin rash during pregnancy, likely or confirmed to be due to ZIKV infection.

After notification, all suspected cases were investigated by local epidemiological surveillance teams and classified as confirmed, probable, inconclusive or unconfirmed. Suspected cases were considered [8]:

- confirmed/probable when they had signs and symptoms consistent with CZS regardless of laboratory confirmation or maternal symptoms.
- unconfirmed if they had compatible clinical symptoms that, after clinical and epidemiological investigation, were attributed to having another cause; for example, microcephaly related to restricted intrauterine growth or genetic diseases.
- inconclusive if there was insufficient information for proper classification.

The Live Births Information System (SINASC) registers data from the Declaration of Live Births. It is legally required document completed by the health worker who assists the delivery [11]. It covers close to 100% of all births in Brazil. We extracted information from SINASC [12] on the date and place of birth, gestational age at birth, birth weight, and newborn sex.

From the Mortality Information System (SIM), which records deaths and provides death certificates (a legally required document that must be completed by the physician who attests the death), we obtained information on the date of death. As of 2011, SIM was estimated to cover more than 96% of all deaths in Brazil [12].

Comparison group: We included a population of live births born in the same period as cases without a linked record of CZS to provide a baseline for the mortality rate comparisons.

All the data were provided by the Brazilian Ministry of Health to the Center of Data and Knowledge Integration for Health (CIDACS) in 2020.

### Linkage process

Data from the 3 sources, SINASC (births), SIM (deaths), and RESP (suspected cases of CZS) were linked. The name of the mother, maternal date of birth or age (when the date of birth of the mother was missing), and place of residence were used as matching variables. Linkage was done at CIDACS in a strict data protection environment and according to ethical and legal rules using CIDACS-RL software. CIDACS-RL is a novel record-linkage tool that applies a combination of indexing and searching algorithms developed in-house to link large-scale administrative datasets [13, 14]. The linkage is processed two by two (SINASC-RESP and SINASC-SIM), then the information from the three datasets are organised in one single database.

### Procedures and definitions

We included all suspected cases of CZS with a complete epidemiological investigation and excluded cases that did not link with a birth registry from the SINASC. Live births who died during the study period were identified by linking SINASC with SIM.

We then classified the suspected cases according to HC and birth weight using the Z scores estimated according to INTERGROWTH 21st standards by sex and gestational age. The newborns were classified as having a normal head circumference (NHC) when between + 2 and -2 standard deviations (SD) from the mean; microcephaly when below two and above or equal three SD of the mean; severe microcephaly when more than 3 SD below the mean; and macrocephaly when more than 2 SD above the mean. According to birth weight, we classified as appropriate for gestational age (AGA) between + 2 and -2 Z SD of the mean; small for gestational age (SGA) more than 2 SD below the mean and large for gestational age (LGA) defined as more than 2 SD above the mean [15]. We also categorised cases according to imaging findings (not abnormal, abnormal compatible with congenital infections, abnormal other) and according to the presence of a maternal rash during pregnancy.

### Statistical analyses

Anthropometry at birth, imaging findings, maternal rash, region and year of birth were categorised in the final epidemiological classification using numbers and percentages for categorical data and means and SD for continuous variables. Differences in characteristics were summarised using the chi-squared test and analysis of variance used to compare means considering a 5% significance level (*p*-value < 0.05).

Latent class analysis was used to cluster unconfirmed cases into classes [16–18]. The resultant classes represented probabilistic groups of patients with similar combinations of characteristics, based on anthropometry at birth (HC and birth weight for gestational age and sex), imaging findings, maternal reported rash, region, and year of birth [16]. Characteristics were described according to each cluster using numbers and percentages and chi-squared tests. Kaplan–Meier curves were plotted, and Cox proportional hazards models were fitted to determine the association of clusters of unconfirmed cases, confirmed cases and the comparison group with mortality up to 36 months. In addition, the association of the individual characteristics was used to fit the model. Mortality in each cluster was assessed using the same Cox proportional hazards models. Data analyses were performed in Stata version 15.0.

This study is reported as per the Reporting of studies Conducted using Observational Routinely-collected Data (RECORD) guideline (Supplementary material 1).

## Results

**Fig. 1:**
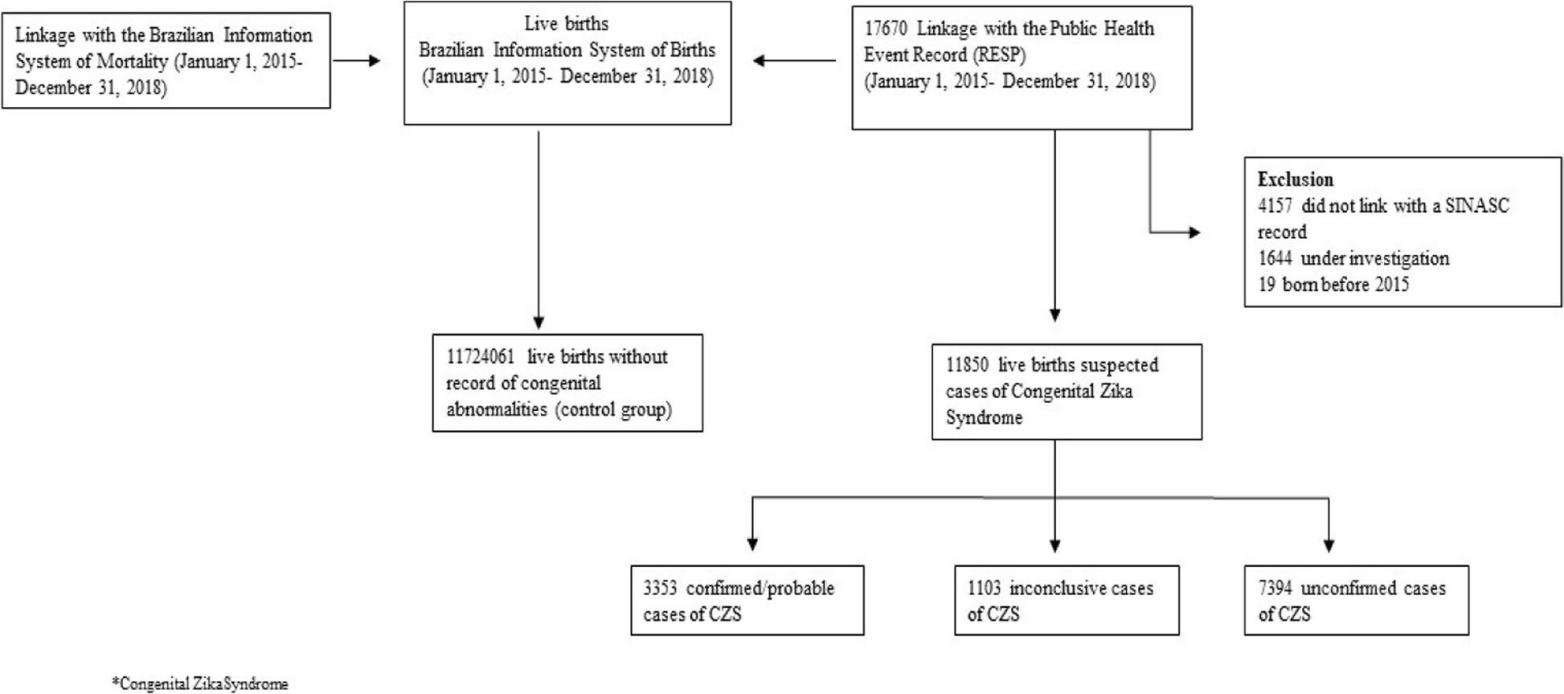
Flowchart study population Brazil, 2015–2018

From January 1, 2015, to December 31, 2018, 17,670 suspected cases of CZS were reported in Brazil; 11,850 (67.1%) of these had a complete investigation and linked with SINASC data and were therefore included in our study. In this final sample, 3353 (28.3%) were confirmed/probable, 1103 (9.3%) were inconclusive, and 7394 (62.4%) were classified as unconfirmed. We also included 11,724,061 live births born in the same period without a linked record of CZS as the comparison group (Fig. 1).

**Fig. 2:**
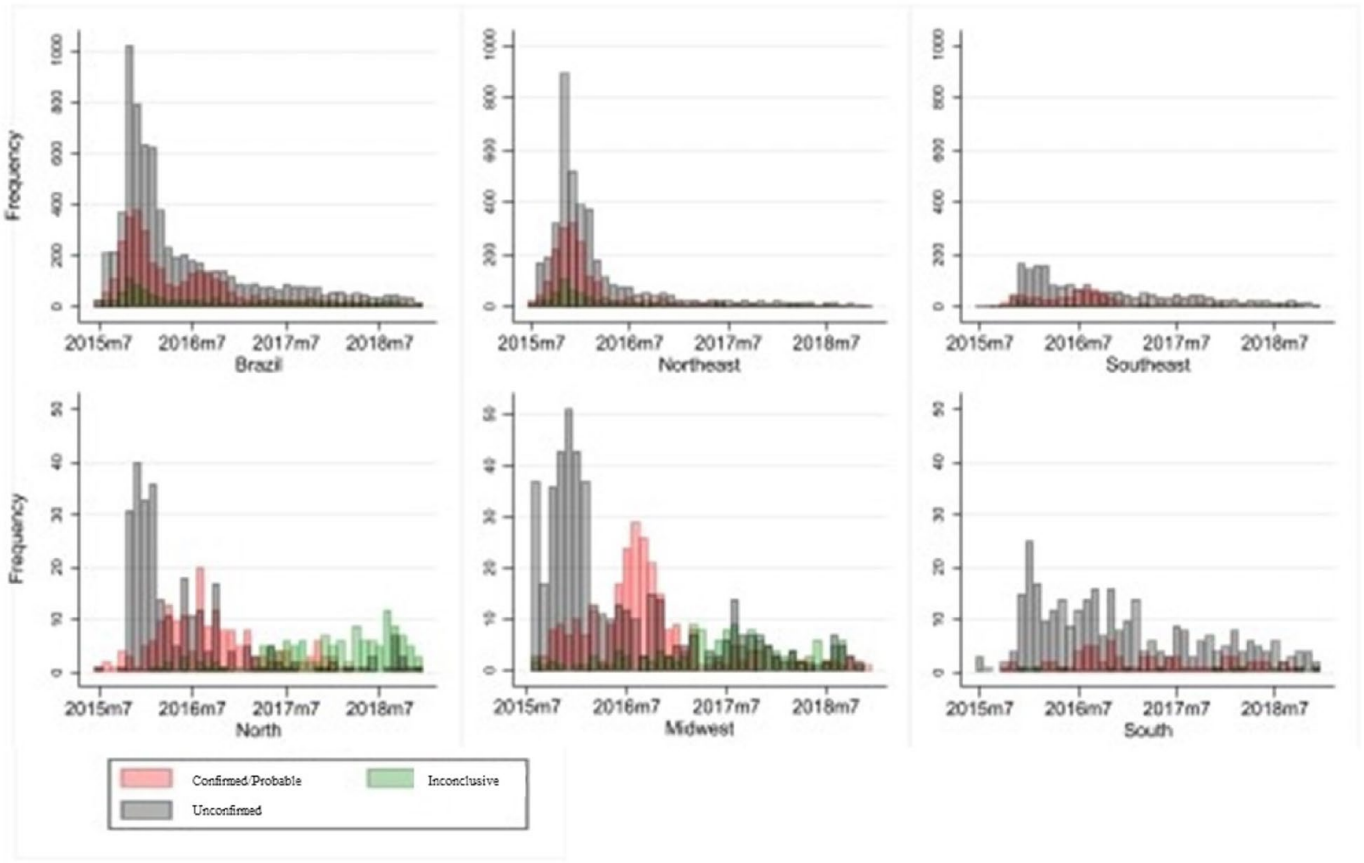
Number of suspected cases of CZS by month and region of birth comparing confirmed, probable, unconfirmed, and inconclusive cases Brazil, 2015–2018. *The Y axis differ because of the small number in some regions

Figure 2 presents the temporal distribution of suspected cases by month of birth and the final classification category for Brazil overall and for 5 regions. Overall, we observed two waves of confirmed/probable cases of CZS occurring from October of 2015 to January 2016 and from July 2016 to October 2016. The first wave of confirmed cases peaked in December of 2015, with nearly 400 cases registered. The peak of the second wave, which occurred in August and September 2016, was about half that size, with fewer than 200 registered cases. The Northeast region had most cases in the first wave, while the second wave occurred mainly in the remaining regions, the Southeast and Midwest in particular. Most confirmed/probable cases notified in the Southeast, North and Midwest regions happened from July to September 2016 whereas cases notified before that were unconfirmed after epidemiological investigation.

Information on HC was available for 86.4% of all suspected cases. The highest percentage of missing data for this variable was observed among inconclusive cases (19.5%) followed by confirmed/probable cases (17.4%). The mean (SD) HC was significantly higher among unconfirmed cases (mean in unconfirmed cases: 31.3 (2.4); and similar in inconclusive cases: 30.1 (2.8) and confirmed/probable cases: 30.1 (3.4). More than 42% of confirmed/probable cases had severe microcephaly, compared to less than 7.5% of unconfirmed cases. In our data, 4.5% of confirmed/probable cases of CZS were born with macrocephaly. Neuroimaging tests were performed for 6623 (55.9%) suspected cases. More than 74.6% of the confirmed/probable cases had a neuroimaging test. These percentages were much lower among inconclusive 37.0% and unconfirmed cases 50.2%. Among confirmed/probable cases, 72.3% (1908) had abnormal findings compatible with congenital infections and 15.7% (415) had other abnormal findings. Among unconfirmed cases, 20.2% (1006) had imaging results with abnormalities (5.3% compatible with congenital infection and 14.9% other abnormalities) and 33.2% among inconclusive cases (15.1% compatible with congenital infection and 18.1% other abnormalities). Maternal rash was reported for nearly 51% of confirmed/probable cases and 18.1% unconfirmed cases. By the end of the study period, 970 suspected cases had died, 404 (12.1%) of confirmed/probable cases, 104 (9.4%) of inconclusive cases, and 462 (6.2%) of unconfirmed cases (Table 1). From the comparison group, 130,471 (1.1%) had died.

**Table 1:** Head circumference and birth weight according to sex and gestational age, imaging finding and rash during pregnancy and mortality comparing confirmed, probable, unconfirmed, and inconclusive CZS cases, Brazil, 2015–2018

	**Confirmed/Probable (3353)**	**Inconclusive (1103)**	**Unconfirmed** **(7394)**	***p* value**
**Head size**				
Mean Head circumference (cm)	30.1 (3.4)	30.1 (2.8)	31.3 (2.4)	
NHC	774 (27.9)	451 (50.8)	4102 (62.3)	< 0.001
Microcephaly	705 (25.5)	238 (26.8)	1815 (27.6)	
Severe Microcephaly	1167 (42.3)	158 (17.8)	493 (7.5)	
Macrocephaly	123 (4.5)	41 (4.6)	174 (2.6)	
Missing	584	215	810	
**Birth weight**				
Mean Birth weight (g)	2639.7 (626.1)	2600.4 (612.4)	2587.1 (570.6)	
AGA	2540 (79.3)	825 (79.1)	5787 (80.5)	0.019
SGA	567 (17.7)	198 (19.0)	1260 (17.5)	
LGA	94 (2.9)	20 (1.9)	140 (1.9)	
Missing	152	60	207	
**Imaging findings**				
No abnormal	177 (6.7)	199 (31.5)	2709 (54.3)	< 0.001
Abnormal compatible with congenital infections	1908 (72.3)	95 (15.1)	263 (5.3)	
Abnormal others	415 (15.7)	114 (18.1)	743 (14.9)	
Not performed	138 (5.2)	223 (35.3)	1276 (25.6)	
Missing	715	472	2403	
**Reported Rash**				
Yes	1377 (51.0)	240 (32.9)	843 (18.1)	< 0.001
No	1324 (49.0)	489 (67.1)	3824 (81.9)	
Missing	652	374	2727	
**Deaths**				
Yes	404 (12.1)	104 (9.4)	462 (6.2)	< 0.001
No	2949 (87.9)	999 (90.6)	6932 (93.8)	

*NHC* Normal head circumference, *AGA* Adequate for gestational age, *SGA* Small for gestational age, *LGA* Large for gestational age

### Latent class analysis

Latent class analysis of unconfirmed cases revealed three distinct clusters based on anthropometry at birth, maternal rash, imaging findings, region, and year of birth. Class 1 is a high morbidity cluster, with frequent severe microcephaly and SGA. It concentrated the highest proportion of unconfirmed cases with abnormal imaging findings (46.7%); mothers from this group were less likely to report a rash during pregnancy compared with the remaining groups. Class 1 includes 16.0% of the unconfirmed population. Unconfirmed cases in class 2 and class 3 tended to have a normal head and birth size. Class 2 cases were concentrated in the Southeast region in 2016. Class 2 included 33.7% of unconfirmed cases. Class 3 cases were less likely to have abnormal findings in imaging tests, and concentrated cases in the Northeast region recorded in 2015 and had the highest proportion of women who reported a maternal rash during pregnancy. Cluster 3 includes 50.3% of the unconfirmed cases (Table 2).

**Table 2:** Head circumference and birthweight according to sex and gestational age, imaging finding and rash during pregnancy and mortality comparing clusters of unconfirmed cases, Brazil, 2015–2018

Variable	Total of unconfirmed cases	Clusters of unconfirmed cases			*p*-value	Missing (%)		
		**Class 1 N = 1183 (16.0%)**	**Class 2 N = 2494 (33.7%)**	**Class 3 N = 3717 (50.3%)**		**Class 1**	**Class 2**	**Class 3**
**Head size**								
NHC	4102 (62.3)	-	1632 (72.9)	2470 (75.7)	< 0.001	257 (10.3)	98 (8.3)	455 (12.2)
Microcephaly	1815 (27.6)	501 (46.2)	603 (27.0)	711 (21.8)				
Severe microcephaly	493 (7.5)	423 (39.9)	-	70 (2.1)				
Macrocephaly	174 (2.6)	161 (14.8)	2 (0.1)	11 (0.3)				
**Birth weight**								
AGA	5787 (80.5)	371 (31.8)	2279 (93.1)	3137 (87.8)	< 0.001	45 (1.8)	17 (1.4)	145 (3.9)
SGA	1260 (17.5)	730 (62.6)	143 (5.8)	387 (10.8)				
LGA	140 (1.9)	65 (5.6)	27 (1.1)	48 (1.3)				
**Imaging findings**								
No abnormal	2709 (54.3)	376 (41.8)	1076 (63.0)	1257 (52.7)	< 0.001			
Abnormal compatible with congenital infections	263 (5.3)	88 (9.8)	133 (7.8)	42 (1.8)		787 (31.6)	284 (24.0)	1332 (35.8)
Abnormal others	743 (14.9)	332 (36.9)	326 (19.1)	85 (3.6)				
Not performed	1276 (25.6)	103 (11.5)	172 (10.1)	1001 (42.0)				
**Reported Rash**								
Yes	843 (18.1)	74 (8.1)	324 (15.1)	445 (27.7)	< 0.001	348 (13.9)	269 (22.7)	2110 (56.8)
No	3824 (81.9)	840 (91.9)	1822 (84.9)	1162 (72.3)				
**Region**								
North	284 (3.8)	26 (2.2)	151 (6.1)	107 (2.9)	< 0.001	-	-	-
Northeast	4205 (56.9)	475 (40.1)	319 (12.8)	3411 (91.8)				
South	315 (4.3)	151 (12.8)	164 (6.6)	-				
Southeast	2118 (28.6)	426 (36.0)	1690 (67.8)	2 (0.1)				
Midwest	472 (6.4)	105 (8.9)	170 (6.8)	197 (5.3)				
**Year**								
2015	2725 (36.8)	107 (9.0)	231 (9.3)	2387 (64.2)	< 0.001	-	-	-
2016	3173 (42.9)	545 (46.1)	1298 (52.0)	1330 (35.8)				
2017	955 (12.9)	323 (27.3)	632 (25.3)	-				
2018	541 (7.3)	208 (17.6)	333 (13.4)	-				

*NHC* Normal head circumference, *AGA* Adequate for gestational age, *SGA* Small for gestational age, *LGA* Large for gestational age

Class 1 characterises the group with concomitant high proportion of microcephaly, SGA and abnormal imaging findings; Class 2 high proportion of normal head size and birth weight and the second highest proportion of abnormal findings in imaging tests; Class 3 high proportion of normal head size and birth weight and low proportion of cases with abnormal findings in imaging tests

**Fig. 3:**
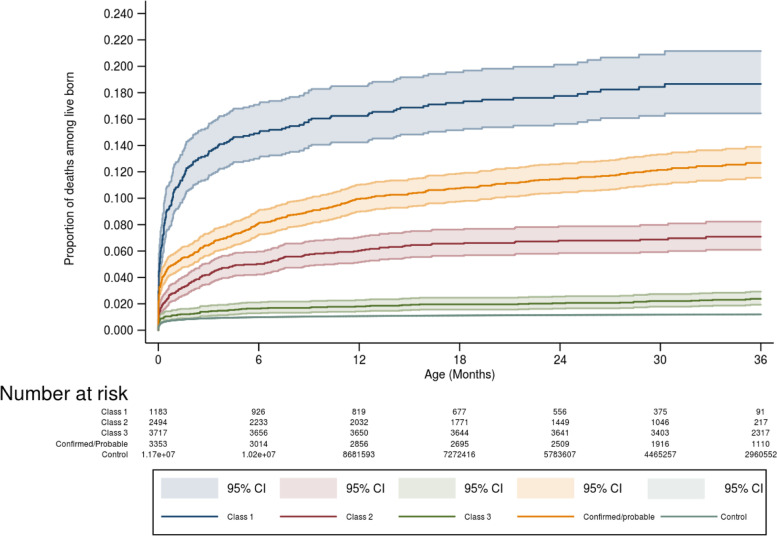
Kaplan–Meier curves showing survival comparing confirmed/probable, Class 1, Class 2 and Class 3 of unconfirmed cases and live births born in the same period without a linked record of CZS (control group), Brazil, 2015–2018. Class 1 characterises the group with concomitant high proportion of microcephaly, SGA and abnormal imaging findings; Class 2 high proportion of normal head size and birth weight and the second highest proportion of abnormal findings in imaging tests; Class 3 high proportion of normal head size and birth weight and low proportion of cases with abnormal findings in imaging tests

Unadjusted all-cause mortality up to 36 months was higher for class 1 than for classes 2 and 3 and even higher than confirmed cases. However, the lowest mortality was observed in class 3, although this was still higher than that observed in the comparison group (Fig. 3). For individual conditions among unconfirmed cases, the worst outcomes were observed in those with abnormal imaging findings: compatible with congenital infection (HR = 12.6; IC95% 8.8–18.0) or other abnormalities (HR = 11.6; IC95% 8.6–15.6) compared with those with no abnormal imaging findings. Severe microcephaly (HR = 8.2; IC95% 6.4–10.6) and macrocephaly (HR = 6.6; (IC95% 4.5–9.7) were also highly associated with mortality, compared to mortality in those with normal head size. Among SGA unconfirmed cases, mortality was 1.9 (HR = 1.9; IC95% 1.5–2.3), and LGA cases was 2.8 (IC95% 1.8–4.5) higher than in unconfirmed AGA cases. Maternal rash was not associated with childhood mortality among unconfirmed cases (Table 3).

**Table 3:** Unadjusted HR for mortality up to 36 months according to clusters of **unconfirmed** cases and individual characteristics, Brazil, 2015–2018

Birth characteristics and imaging findings	Deaths per 1000 person-Years	Unadjusted (HR) (95% CI)
**Final classification and cluster group**		
Class 1	104.2 (90.9–119.4)	9.0 (7.0–11.5)
Class 2	33.1 (28.4–38.5)	3.1 (2.4–4.1)
Class 3	8.2 (6.6–10.1)	Ref
**Head size**		
NHC	12. (10.8–15.2)	Ref
Microcephaly	18.6 (14.9–23.2)	1.3 (1.0–1.8)
Severe Microcephaly	129.3 (107.4–155.6)	8.2 (6.4–10.6)
Macrocephaly	114.0 (81.0–160.4)	6.6 (4.5–9.7)
**Birth weight**		
AGA	21.2 (18.9–23.7)	Ref
SGA	43.3 (36.2–51.9)	1.9 (1.5–2.3)
LGA	66.3 (42.3–103.9)	2.8 (1.8–4.5)
**Imaging findings**		
No abnormal	9.5 (7.4–12.3)	Ref
Abnormal compatible with congenital infections	148.9 (116.3–190.7)	12.6 (8.8–18.0)
Abnormal others	138.2 (118.6–160.9)	11.6 (8.6–15.6)
Not performed	14.8 (11.2–19.6)	1.7 (1.2–2.5)
**Reported Rash**		
Yes	26.2 (20.0–34.2)	Ref
No	34.7 (30.9–39.0)	1.2 (0.9–1.6)

*NHC* Normal head circumference, *AGA* Adequate for gestational age, *SGA* Small for gestational age, *LGA* Large for gestational age

Class 1 characterises the group with concomitant high proportion of microcephaly, SGA and abnormal imaging findings; Class 2 high proportion of normal head size and birth weight and the second highest proportion of abnormal findings in imaging tests; Class 3 high proportion of normal head size and birth weight and low proportion of cases with abnormal findings in imaging tests

## Discussion

This nationwide study of all suspected cases of CZS from 2015 to 2018 confirmed that although microcephaly is the main feature of the syndrome, in some confirmed cases, newborns had a normal head size, and some even had macrocephaly at birth. Among unconfirmed cases, we identified three distinct clusters with different mortality trajectories. Class 1, which combined the highest proportion of abnormal head size and imaging findings, had the highest mortality rate. The lowest mortality rate was observed in Class 3; however, it was still higher than that observed in the comparison group of live births from the same period. Each characteristic (except maternal rash) was associated with a unique and significant mortality risk; the greatest was for those with abnormal imaging findings, who were over 11 times more likely to die than those with no abnormalities detected, and for those with severe microcephaly and macrocephaly, who were over six times more likely to die than those with normal head size.

The temporal analyses confirmed the previous report of two waves of CZS cases [19] and revealed that the Northeast region drove the first and highest wave, while the second one was propelled by the Southeast and Midwest regions. This reflects the ZIKV dispersion in the country, where the Northeast is the point of introduction and dissemination of the virus [20].

Previous reports using routinely collected data from Brazil limited their analyses to cases with microcephaly because knowledge about the phenotype of CZS was restricted to this condition at the beginning of the epidemic [9]. More recent studies have provided evidence that CZS is a syndrome with a broad spectrum of presentations [10]. Although microcephaly was the main feature observed in our data, expected given the case definition, we identified confirmed cases among infants with a normal head circumference and some with macrocephaly. Although macrocephaly is a clinical symptom of heterogeneous causes, it should alert clinicians to potentially enlarged ventricles. This is consistent because hydrocephalus is a complication of CZS in at least a proportion of cases, as described by van der Linden et al*.* (2019) [21].

To our knowledge, this is the first study to investigate the medium-term mortality risk in all suspected cases of CZS, including those unconfirmed. Confirmed cases had almost two times higher mortality when compared with all unconfirmed cases; unconfirmed cases had higher mortality than the control group. Unconfirmed cases were notified for a reason; that reason was likely to have been a different diagnosis or a CZS that did not meet current, more strict criteria. Unconfirmed cases with severe clinical features, a combination of abnormal head and birth size and abnormal imaging findings, were concentrated in Class 1. Class 1 unconfirmed cases had the highest mortality, almost two times higher than that of confirmed CZS cases and nine times higher than the group of unconfirmed cases with no abnormal findings in imaging (cluster 3). We believe Class 1 cases are likely to suffer from severe malformations or from severe congenital infections with a high mortality rate; a small minority may have been atypical CZS. Class 2 unconfirmed cases had the second-highest proportion of abnormal imaging findings among unconfirmed cases. Their mortality was lower than that of confirmed/probable CZS cases but the second-highest mortality among unconfirmed case. We suggest that they include some CZS unusual presentation, less severe forms of TORCH and some normal newborns with just small head circumference. Most of the cases in Class 3 occurred early in the Zika epidemic, concentrated in the country’s Northeast region among women who had reported a rash, and their mortality was just a little higher than controls. We suggest some of these cases resulted from zealous reporting before the CZS was well understood and the criteria refined, early in the epidemic, with a few cases of atypical CZS or mild TORCH.

Identifying specific survival trajectories for clusters of unconfirmed cases has potentially important repercussions. It highlights that a group of unconfirmed cases has similar healthcare needs to confirmed cases. At the moment, there is no recommendation for the monitoring and care of unconfirmed cases like there is for confirmed cases of CZS [8]. Therefore, the group of unconfirmed cases is less likely to receive appropriate follow-up care, which may even contribute to their high mortality rates. For individual factors, abnormal imaging findings and severe microcephaly were the two individual characteristics associated with the poorest prognoses.

This study has strengths and limitations. It was conducted using all suspected cases of CZS. It applied latent class analysis to provide further insight into the heterogeneous group of unconfirmed cases and their mortality differentials. Using this method, we were able to model complex characteristics and their association with survival. Despite these strengths, there were study limitations related to the study being based on routine data. First, it relied on the accuracy of the registry of data and the limited number of variables collected routinely. Second, we observed a relatively high proportion of missing data, which could have biased the estimates. Third, at the beginning of the epidemic, the case definition of suspected cases only included newborns with microcephaly, as a result, newborns without microcephaly but with features of CZS would not have been reported biasing temporal measures. Fourth, the linkage process could have introduced a classification bias due to a linkage error. However, it is probably non-differential among the clusters of unconfirmed cases and therefore unlikely to introduce bias. The proportion of cases with incomplete investigation and those without link with SINASC limited our analysis to 67% of CZS suspected cases. It may have introduced bias to our results. Finally, the study did not include any clinical follow-up of notified cases.

In conclusion, based on our analysis of data on all suspected cases of CZS we add to the expanding understanding of this condition. Although microcephaly is the main feature of CZS, we confirm that some cases had a normal head size, and a few had macrocephaly at birth. We recommend a clinical review of records to expand the clinical definition of CZS presentations. Unconfirmed cases are clearly at increased mortality risk, and that maybe because they are atypical CZS cases, or because of an alternative diagnosis. We defined 3 classes within unconfirmed cases, with a different presentation at birth and specific mortality trajectories. We observed that specific characteristics, such as abnormal head size and imaging findings, seem to be responsible for the higher mortality risk among unconfirmed cases. We suggest that this information be further used to develop predictive models to discriminate against those more likely to die. The final aim is to optimise interventions and enhance resource allocation for those most in need regardless of the final diagnoses, or epidemiological classification of CZS suspected cases.

## Supplementary Information

**Additional file 1.** The RECORD statement.

## Data Availability

The data that support the findings of this study are available from Brazilian Ministry of Health, but restrictions apply to the availability of these data, which were used under license for the current study, and so are not publicly available. Data are however available from the authors upon reasonable request and with permission of the Brazilian Ministry of Health. For more information contact CIDACS curation team cidacs.curadoria@fiocruz.br.
